# Forecasting Kazakhstan’s regional population aged 6–17 years to 2030: cohort validation and uncertainty analysis

**DOI:** 10.3389/fpubh.2026.1903413

**Published:** 2026-09-07

**Authors:** Nurlan Jainakbayev, Lyazat Orakbay, Karlygash Zhubanysheva, Madina Kamalieva, Zulfiya Elzhanova, Nurlan Baisynov, Firuza Numanova

**Affiliations:** 1 Department of Public Health, Non-profit Educational Institution “Kazakhstan-Russian Medical University”, Almaty, Kazakhstan; 2 Department of Neonatology, Non-profit Educational Institution “Kazakhstan-Russian Medical University”, Almaty, Kazakhstan; 3 Department of Scientific Work, Non-profit Educational Institution “Kazakhstan-Russian Medical University”, Almaty, Kazakhstan

**Keywords:** ARIMA, cohort projection, demographic forecasting, empirical uncertainty, ETS, Kazakhstan, population aged 6–17 years, rolling-origin validation

## Abstract

**Background:**

Regional forecasts of the population aged 6–17 years can provide demographic denominators for education and school health planning, but they do not directly estimate health-service or workforce requirements. This study aimed to forecast the population aged 6–17 years across Kazakhstan’s 20 territorial units through 2030 and to compare the historical accuracy of a focal age-shift cohort model with ARIMA and ETS benchmarks.

**Methods:**

Official aggregated population data for 2015–2025 were analyzed for 20 territorial units by region, sex, locality type, and single year of age. The cohort, ARIMA, and ETS models were evaluated on the same five expanding-window one-step targets for 2021–2025. Empirical uncertainty in cohort-transition ratios was assessed using transition-year block bootstrap resampling; coefficient sensitivity and exploratory migration-effect stress tests were also examined.

**Results:**

The cohort model had the lowest rolling-origin RMSE in 17 of 20 territories, compared with two for ETS and one for ARIMA. By 2030, the largest point-estimate increases were projected for Astana, Shymkent, Almaty city, and Mangystau Region, while the largest decline was projected for North Kazakhstan Region. Empirical uncertainty intervals supported growth in eight territories and decline in two; the direction remained uncertain in ten.

**Conclusion:**

Under the primary validation design, the cohort model had the lowest historical forecast error by RMSE in most territories. The projections quantify a demographic denominator; translating them into staffing, service, or infrastructure requirements requires additional data on morbidity, coverage, utilization, accessibility, productivity, and existing capacity.

## 1 Introduction

For age-defined target populations, regional forecasting is most informative when it represents both population size and age structure. Aggregate time-series models extend historical totals, whereas cohort methods explicitly follow the movement of age groups through time and are therefore well suited to short- and medium-term projections when the relevant cohorts are already observed (1–5). For the population aged 6–17 years, single-year age data make it possible to model entry into and exit from the target interval without forecasting births over a five-year horizon.

Kazakhstan presents a particularly relevant setting because demographic dynamics differ markedly across its regions and three cities of republican significance. Previous work has linked demographic growth to pressure on educational infrastructure (6), while official statistics and national demographic forecasts document substantial territorial heterogeneity (7–9). However, regional forecasts covering the full 6–17-year age range have rarely compared age-structured and aggregate models on identical historical forecast origins or quantified uncertainty arising from recent cohort transitions.

For school health planning, the projected population aged 6–17 years should be treated as a demographic denominator rather than a direct estimate of need. A demographic forecast can therefore inform, but should not substitute for, a health needs assessment (10). Within this boundary, the projected population can quantify the population potentially covered by school-based preventive examinations, screening, vaccination-related activities, and health-promotion programs (11–16). Converting population counts into service, staffing, or infrastructure requirements requires additional information on morbidity, service standards, coverage targets, utilization, school locations, travel time, workforce productivity, and existing capacity (17–21).

The aim of this study was to forecast the population aged 6–17 years in 20 territorial units of Kazakhstan through 2030 using a focal age-shift cohort model; to compare its historical forecast accuracy directly with ARIMA and ETS benchmarks under common rolling-origin and fixed-holdout designs; and to characterize empirical transition-ratio, model-specification, and exploratory migration-effect uncertainty. The analytical hierarchy was defined as follows: the cohort model was the focal forecast; ARIMA and ETS were historical validation benchmarks; the block bootstrap quantified variability in observed transition years; coefficient sensitivity tested model specification; and migration scenarios were exploratory stress tests rather than probabilistic migration forecasts.

## 2 Materials and methods

### 2.1 Study design, scope, and data sources

This was an applied regional demographic forecasting study based on official aggregated statistics. The outcome was the annual number of residents aged 6–17 years in each of 20 territorial units (17 regions and Astana, Almaty city, and Shymkent). The study did not analyze morbidity, service utilization, preventive-service coverage, workforce, infrastructure, or costs; consequently, it did not estimate health-service requirements.

The primary source was the Demographic Statistics dynamic tables of the Bureau of National Statistics of the Agency for Strategic Planning and Reforms of the Republic of Kazakhstan (7), accessed on April 29, 2026. The population table provided counts by calendar year, region, sex, locality type, and single year of age. Data for ages 0–17 years and calendar years 2015–2025 were retained. Total regional population and net migration for 2021–2024 were used only for consistency checks and exploratory migration-effect stress tests.

Territorial labels were harmonized to the current 20-unit classification. The historical values for regions established in 2022 were taken as reported in the official retrospective series; no author-generated redistribution of earlier observations was performed. For the three cities of republican significance, the absence of a rural stratum was treated as structural rather than missing.

### 2.2 Analytical dataset and quality control

The analytical grain was year × region × sex × locality type × single year of age. Duplicate keys, missing years, non-finite or negative counts, incomplete age coverage, and conflicting repeated totals were checked before modeling. Regional totals for ages 6–17 years were obtained by summing the corresponding single-year age cells across sex and locality. The final observed series contained 11 annual values for every territorial unit, from 2015 through 2025.

The forecast horizon ended in 2030. With 2025 as the baseline, all cohorts that would be aged 6–17 years in 2030 were already observed at ages 1–12 years in 2025. A five-year projection therefore did not require assumptions about future births.

### 2.3 Integrated analytical framework

The analysis had one focal model and four complementary components. First, the age-shift cohort model produced the primary 2026–2030 forecast. Second, ARIMA and ETS models served as aggregate time-series benchmarks in historical validation. Third, a transition-year block bootstrap quantified empirical variability associated with the five observed transition years. Fourth, alternative coefficient windows and summary statistics assessed specification sensitivity. Finally, migration-effect scenarios were treated as exploratory stress tests. Future forecasts from different model families were not averaged, and scenario results were not interpreted as additional deterministic predictors.

### 2.4 Focal age-shift cohort model

For region r, sex s, locality type u, age a, and year t, the annual cohort-transition ratio was defined as:


$$K(r,s,u,a,t)=\frac{\left(r,s,u,a+1,t+1\right)}{N(r,s,u,a,t)}$$

The ratio therefore followed the same birth cohort from age *a* in year *t* to age *a* + 1 in year *t* + 1. It incorporated mortality, migration, boundary or reporting effects, and other changes only insofar as these were reflected in observed cohort movement. For the final forecast, the coefficient for each region–sex–locality–destination-age cell was the median of the five ratios with origin years 2020–2024. No arbitrary lower or upper truncation was applied.

A hierarchical fallback to region–age and then national age-specific ratios was specified for unavailable cells. In the final dataset, all 1,258 required coefficient cells were estimable at the full region–sex–locality–destination-age level, so no fallback was used. Starting from the 2025 population, coefficients were applied recursively for 2026–2030. Regional totals were calculated as:


S(r,t)=Σ[a=6…17]N(r,a,t)

Absolute and percentage changes in 2030 were calculated relative to the observed 2025 value.

### 2.5 Historical validation and benchmark models

The primary validation placed the cohort, ARIMA, and ETS models on identical expanding-window one-step forecast origins. Data through 2020 were used to predict 2021, and the training window was then expanded by 1 year at each origin until data through 2024 were used to predict 2025. Thus, each model produced five forecasts per territorial unit. At each origin, cohort-transition coefficients were re-estimated from the five most recent annual transitions available strictly before the target year. ARIMA models were fitted with automatic order selection, and ETS models with automatic error, trend, and seasonality selection; annual series had frequency one.

A secondary fixed holdout used 2015–2023 for training and 2024–2025 for testing. For the cohort model, transition coefficients were estimated from the five most recent transitions within the training period and then held fixed for the two-step projection. Mean absolute error (MAE), root mean square error (RMSE), mean absolute percentage error (MAPE), and mean error were calculated by region and model. The lowest RMSE defined the best-performing model; MAE and MAPE were secondary measures. The rolling-origin design was primary because it used five common forecast origins, whereas the fixed holdout contained only two test years.

### 2.6 Empirical transition-ratio uncertainty

Uncertainty around the focal cohort projection was evaluated using a nonparametric transition-year block bootstrap with 10,000 simulations and random-number seed 20,260,315. In each simulation, five of the observed transition years with origin years 2020–2024 were sampled with replacement. The same sampled annual blocks were applied across all coefficient cells, preserving cross-cell dependence within each transition year. Cell-specific medians of the resampled ratios were then projected recursively from 2025 through 2030.

For each region and year, the 2.5th and 97.5th percentiles of simulated totals formed a 95% empirical transition-ratio uncertainty interval. The deterministic point estimate remained the projection based on the original five-year median. The 10,000 repetitions were used to stabilize the empirical percentile estimates; they do not increase the effective amount of historical information, which remains limited to five observed annual transition blocks. These intervals therefore quantify sensitivity to recent transition-ratio variability rather than all sources of forecast uncertainty. They are not formal all-source prediction intervals and do not include model-form uncertainty, future structural breaks, abrupt migration shocks, census or territorial revisions, or policy changes.

### 2.7 Coefficient sensitivity and migration-effect stress tests

Coefficient sensitivity was assessed under four specifications: the median for 2020–2024 (focal), the mean for 2020–2024, the median for 2022–2024, and the median for 2018–2024. For each region, the 2030 sensitivity range was the maximum minus the minimum projection across these four specifications.

Migration effects were already embedded in observed cohort-transition ratios and were not added as a separate predictor to the focal model. For exploratory stress testing, the signed annual school-age migration proxy was calculated as:


$$\begin{array}{l} \mathrm{Mr}=\text{mean}(\text{NetMigrationr},2021–2024)\\ \times \text{mean}(\text{Share aged}\;6–17r,2021–2024) \end{array}$$

where net migration and the share aged 6–17 years were averaged over 2021–2024. The focal projection represented continuation of the embedded historical effect (1×). An attenuated alternative (0×) and an amplified alternative (2×) were calculated for horizon h as:


$$\begin{array}{l} \text{Natt}(r,t+h)=\text{Nbase}(r,t+h)-\mathrm{hMr};\\ \text{Namp}(r,t+h)=\text{Nbase}(r,t+h)+\mathrm{hMr} \end{array}$$

These stylized alternatives assumed that school-age migration was proportional to the population share aged 6–17 years. They were used only to show directional sensitivity and were not interpreted as age-specific or probabilistic migration forecasts.

### 2.8 Statistical software and ethics

Data processing, modeling, validation, and visualization were performed in R version 4.5.2 using readxl, dplyr, tidyr, forecast, ggplot2, and writexl. The analysis used secondary aggregated data and included no individual-level or identifiable information; individual informed consent was not applicable.

## 3 Results

### 3.1 Data coverage and model inputs

The final observed dataset contained complete annual regional series for 2015–2025, with 20 territorial units and no conflicting duplicate keys. The observed national population aged 6–17 years totaled 4,491,452 in 2025. All 1,258 required region–sex–locality–destination-age coefficient cells were estimable at the full stratification level without fallback. Median transition coefficients ranged from 0.932 to 1.053, with a median of 0.996.

### 3.2 Focal forecasts and empirical uncertainty

**Figure 1:**
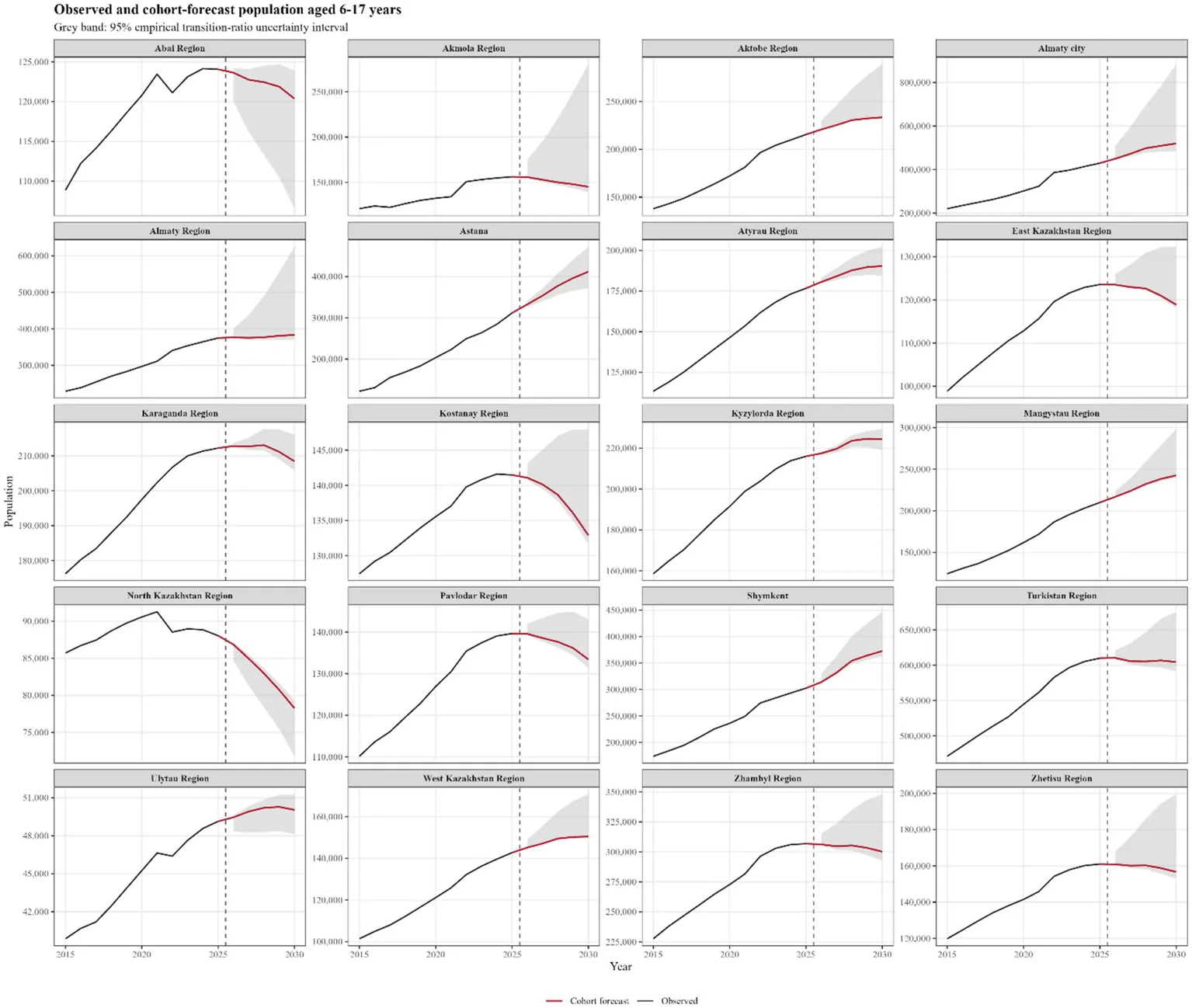
Observed population aged 6–17 years in 2015–2025 and focal cohort forecasts for 2026–2030 by territorial unit. The black line denotes observed values, the red line denotes the focal cohort point forecast, and the vertical dashed line separates the observed and forecast periods. The gray band is the 95% empirical transition-ratio uncertainty interval derived from 10,000 transition-year block-bootstrap simulations; it is not a comprehensive all-source prediction interval.

The point forecasts indicated the largest relative increases between 2025 and 2030 in Astana (+32.14%), Shymkent (+23.18%), Almaty city (+21.09%), and Mangystau Region (+15.54%). The largest point-estimate declines were projected for North Kazakhstan Region (−11.12%), Akmola Region (−7.09%), and Kostanay Region (−6.06%) (Table 1; Figure 1).

**Table 1:** Observed 2025 population and focal cohort projection for 2030.

Territorial unit	Observed 2025	Forecast 2030	Absolute change	Change (%)
Astana	311,894	412,128	+100,234	+32.14%
Shymkent	302,515	372,632	+70,117	+23.18%
Almaty city	429,223	519,727	+90,504	+21.09%
Mangystau Region	210,029	242,669	+32,640	+15.54%
Aktobe Region	215,491	233,445	+17,954	+8.33%
Atyrau Region	176,706	190,441	+13,735	+7.77%
West Kazakhstan Region	142,704	150,421	+7,717	+5.41%
Kyzylorda Region	215,996	224,399	+8,403	+3.89%
Almaty Region	374,867	383,308	+8,441	+2.25%
Ulytau Region	49,140	50,043	+903	+1.84%
Turkistan Region	609,959	604,463	−5,496	−0.90%
Karaganda Region	212,218	208,429	−3,789	−1.79%
Zhambyl Region	306,794	300,197	−6,597	−2.15%
Zhetisu Region	160,993	156,665	−4,328	−2.69%
Abai Region	124,062	120,364	−3,698	−2.98%
East Kazakhstan Region	123,566	118,879	−4,687	−3.79%
Pavlodar Region	139,653	133,422	−6,231	−4.46%
Kostanay Region	141,471	132,892	−8,579	−6.06%
Akmola Region	156,128	145,059	−11,069	−7.09%
North Kazakhstan Region	88,043	78,254	−9,789	−11.12%

The focal point forecast used median transition coefficients for origin years 2020–2024. Values are rounded to whole persons.

**Figure 2:**
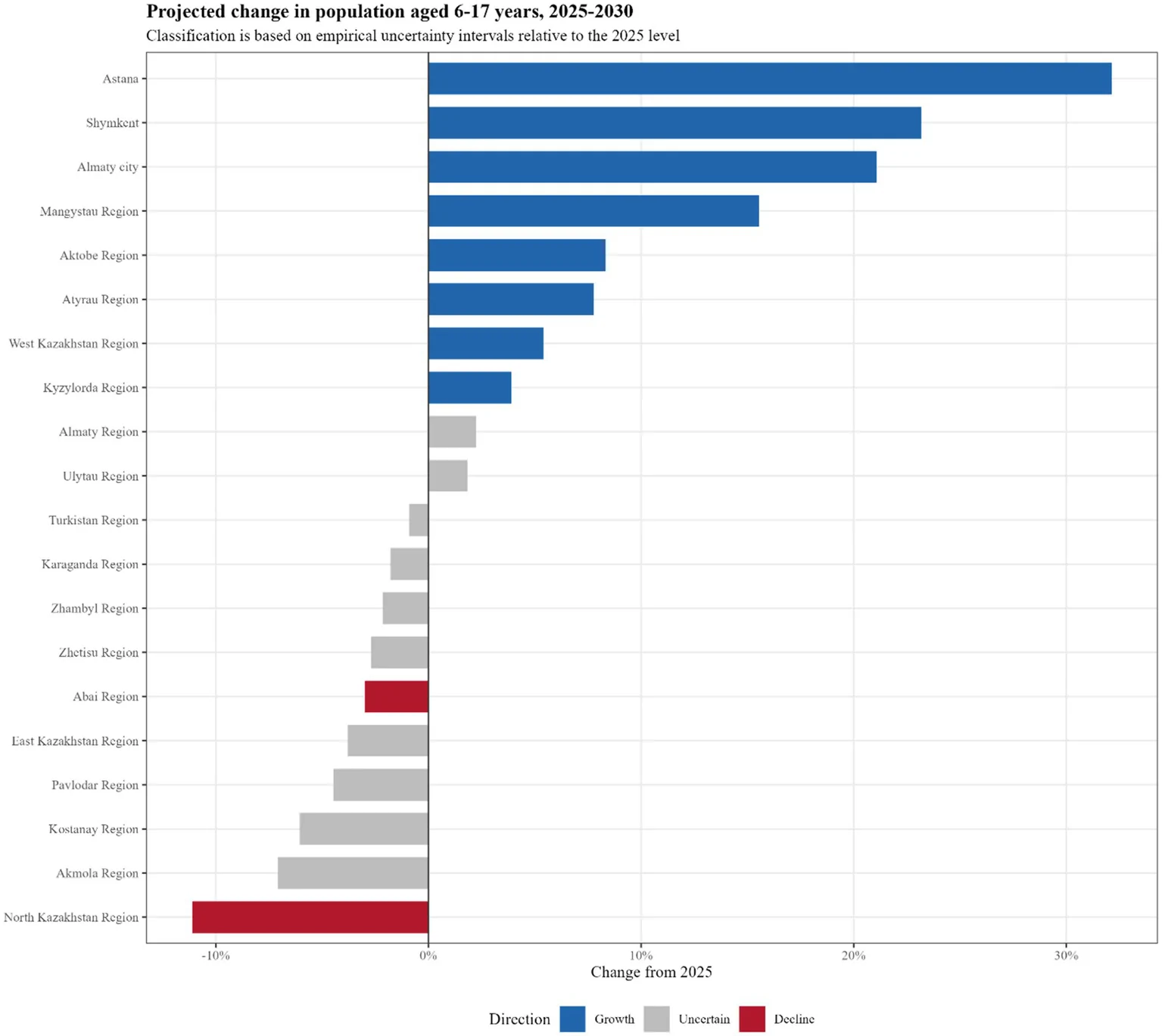
Projected percentage change in the population aged 6–17 years from 2025 to 2030. Colors show whether the empirical transition-ratio uncertainty interval lies above the observed 2025 value (growth), below it (decline), or includes it (uncertain).

The empirical intervals supported growth in eight territories: Astana, Shymkent, Almaty city, Mangystau, Aktobe, Atyrau, West Kazakhstan, and Kyzylorda. Decline was supported in Abai and North Kazakhstan Regions. Direction remained uncertain in the other ten territories because their intervals included the observed 2025 level (Table 2; Figure 2). The widest 2030 intervals occurred in Almaty city, Almaty Region, Akmola Region, Astana, and Shymkent.

**Table 2:** Focal 2030 forecast and empirical transition-ratio uncertainty.

Territorial unit	2025	2030 point	Lower 95% limit	Upper 95% limit	Change (%)	Direction
Astana	311,894	412,128	372,212	472,627	+32.14%	Growth
Shymkent	302,515	372,632	362,364	446,938	+23.18%	Growth
Almaty city	429,223	519,727	483,720	883,878	+21.09%	Growth
Mangystau Region	210,029	242,669	239,363	298,078	+15.54%	Growth
Aktobe Region	215,491	233,445	231,806	288,941	+8.33%	Growth
Atyrau Region	176,706	190,441	184,534	202,316	+7.77%	Growth
West Kazakhstan Region	142,704	150,421	149,507	170,728	+5.41%	Growth
Kyzylorda Region	215,996	224,399	219,176	229,246	+3.89%	Growth
Almaty Region	374,867	383,308	369,314	625,123	+2.25%	Uncertain
Ulytau Region	49,140	50,043	48,114	51,277	+1.84%	Uncertain
Turkistan Region	609,959	604,463	591,284	675,660	−0.90%	Uncertain
Karaganda Region	212,218	208,429	205,850	216,107	−1.79%	Uncertain
Zhambyl Region	306,794	300,197	292,710	348,010	−2.15%	Uncertain
Zhetisu Region	160,993	156,665	152,833	199,485	−2.69%	Uncertain
Abai Region	124,062	120,364	106,535	123,910	−2.98%	Decline
East Kazakhstan Region	123,566	118,879	118,090	132,321	−3.79%	Uncertain
Pavlodar Region	139,653	133,422	131,386	143,037	−4.46%	Uncertain
Kostanay Region	141,471	132,892	131,657	147,899	−6.06%	Uncertain
Akmola Region	156,128	145,059	139,284	280,386	−7.09%	Uncertain
North Kazakhstan Region	88,043	78,254	71,787	79,272	−11.12%	Decline

The limits are the 2.5th and 97.5th percentiles of 10,000 transition-year block-bootstrap simulations. Direction is classified relative to the observed 2025 level. These are empirical transition-ratio uncertainty intervals, not comprehensive all-source prediction intervals.

### 3.3 Comparative historical validation

**Figure 3:**
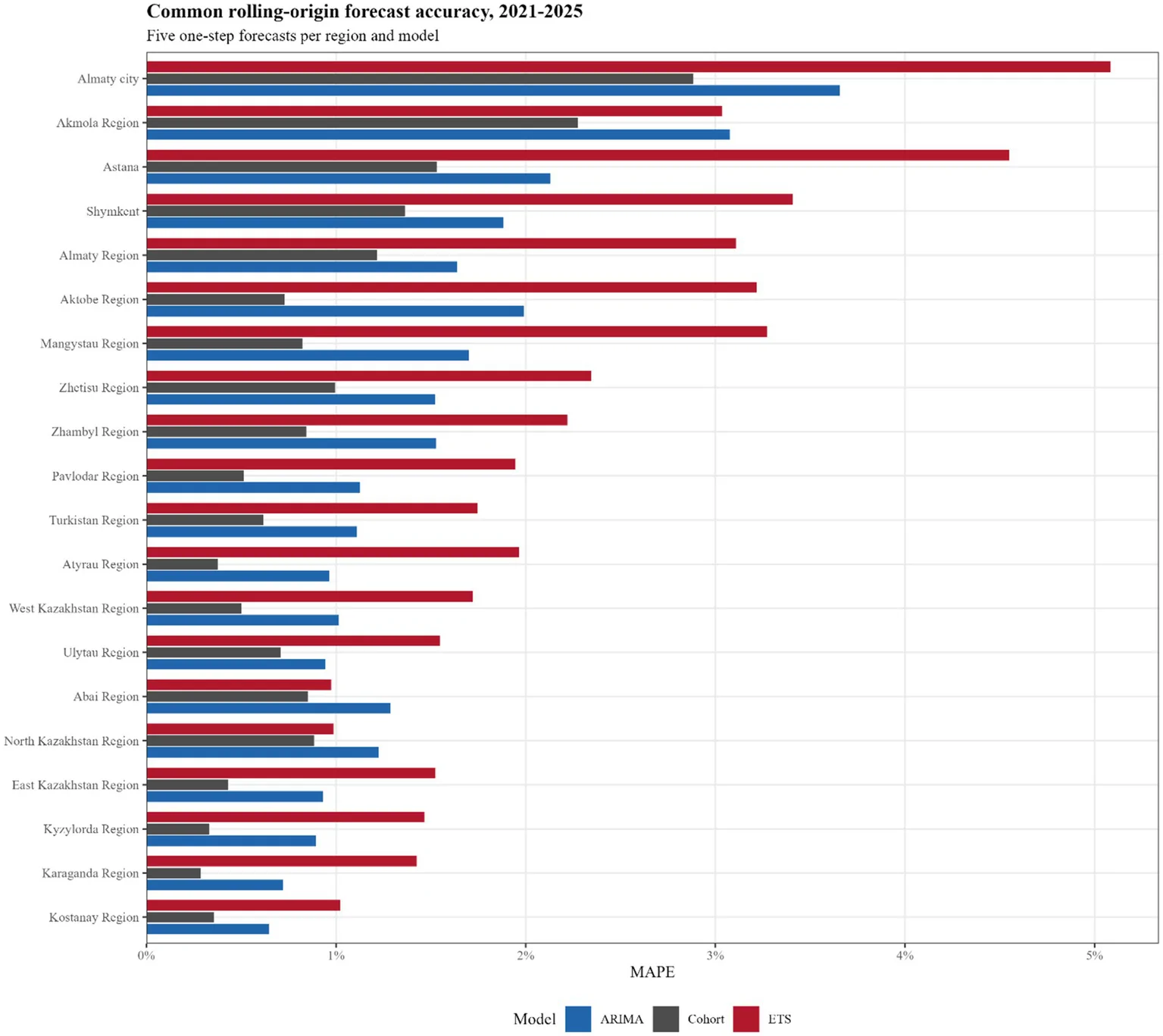
Common rolling-origin forecast accuracy for the cohort, ARIMA, and ETS models, 2021–2025. Bars show regional MAPE calculated from five one-step forecasts per territorial unit and model. Model ranking was based on RMSE.

In the common rolling-origin evaluation, the cohort model had the lowest RMSE in 17 territories, ETS in two (Abai and North Kazakhstan), and ARIMA in one (Akmola). Median regional MAPE was 0.77% for the cohort model, 1.26% for ARIMA, and 1.96% for ETS (Table 3; Figure 3). In the fixed 2024–2025 holdout, the cohort model had the lowest RMSE in all 20 territories and a median regional MAPE of 0.31%, compared with 2.04% for ARIMA and 1.98% for ETS. Model selection agreed between the two validation schemes in 17 territories.

**Table 3:** Comparative historical forecast accuracy.

Model	Rolling-origin median regional MAPE	Best RMSE, rolling (n/20)	Fixed-holdout median regional MAPE	Best RMSE, holdout (n/20)
Cohort	0.77%	17	0.31%	20
ARIMA	1.26%	1	2.04%	0
ETS	1.96%	2	1.98%	0

Rolling-origin validation comprised five common one-step targets (2021–2025). The fixed holdout used 2015–2023 for training and 2024–2025 for testing. Region-specific MAE, RMSE, MAPE, mean error, and model rankings are reported in Supplementary Tables S2A–S2C.

### 3.4 Specification sensitivity and migration-effect stress tests

The largest ranges across the four coefficient specifications were observed in Almaty city (63,671 children), Almaty Region (25,117), Akmola Region (20,408), Shymkent (20,123), and Astana (16,740). Exploratory migration-effect stress-test ranges were largest in Astana (92,870), Almaty city (66,606), Turkistan Region (65,009), Zhambyl Region (31,830), and Zhetisu Region (20,362). These ranges describe sensitivity to stylized assumptions and are not probabilistic migration forecasts.

## 4 Discussion

This study developed and historically evaluated a regional demographic forecasting approach for Kazakhstan’s population aged 6–17 years. Three findings are central. First, the age-shift cohort model had the lowest rolling-origin RMSE in 17 of 20 territories and the lowest fixed-holdout RMSE in all 20, suggesting that recent single-year age structure contained predictive information not captured by aggregate trend extrapolation in these validation periods. Second, point forecasts showed continued growth concentrated in Astana, Shymkent, Almaty city, and Mangystau Region, while the strongest decline was projected for North Kazakhstan Region. Third, the uncertainty-supported classification was more conservative than the point estimates: growth was supported in eight territories, decline in two, and direction remained uncertain in ten.

The comparative validation addresses the principal methodological question: the focal cohort model was evaluated on the same observed historical targets as ARIMA and ETS rather than being justified by differences among future forecasts. Its median regional MAPE was 0.77%, compared with 1.26% for ARIMA and 1.96% for ETS. This pattern is substantively plausible because the cohort model represents entry into and exit from the 6–17-year interval, whereas the time-series models extrapolate aggregate momentum. Nevertheless, the annual series was short. The earliest rolling forecast used six annual observations for model fitting, and the fixed holdout contained only two test years. The results support the use of the cohort model as the focal forecast for the present data and horizon, but they do not establish universal superiority across longer series, alternative periods, or different structural regimes.

Regional patterns should be interpreted as demographic trajectories rather than causal decompositions. Growth in the largest cities is consistent with urban concentration and mobility described in national forecasts and urban studies (8, 9, 22, 23), while differing trajectories across other regions are compatible with heterogeneous age structure and population redistribution. However, the cohort-transition ratios combine survival, migration, reporting changes, boundary effects, and other influences. Accordingly, this is an applied cohort-transition model rather than a full cohort-component projection with separately modeled fertility, mortality, and migration, and it cannot estimate the separate causal contribution of those components to a regional forecast.

The block-bootstrap results materially changed the interpretation of certainty. Wide 2030 intervals in Almaty city, Almaty Region, Akmola Region, Astana, and Shymkent reflected the influence of resampling different recent transition-year blocks and compounding transition ratios over five forecast steps. Only five annual blocks underlay the resampling distribution; increasing the number of simulations reduced Monte Carlo error but did not add historical information. For this reason, the intervals are described as empirical transition-ratio uncertainty intervals rather than comprehensive prediction intervals. They may still underestimate structural uncertainty arising from territorial or census revisions, migration shocks, policy changes, model-form uncertainty, or future departures from recent cohort behavior.

The migration scenarios require similar restraint. The proxy assumed that the school-age component of net migration was proportional to the overall regional population share aged 6–17 years. Migration is age-selective and related to family composition, so this proportionality assumption may not hold. The 0 × and 2 × scenarios are therefore exploratory historical migration-effect stress tests showing how results could shift if the migration effect embedded in recent cohort transitions weakened or strengthened; they are not age-specific or probabilistic forecasts of future child migration. The largest scenario ranges identify territories where age-specific migration data would be especially valuable for future model refinement.

For public health planning, these projections should be used as demographic denominators (10, 17–21). They can indicate where the population base potentially covered by school-based preventive services may increase, remain broadly stable, or decline (11–16, 19–21). They cannot by themselves determine the number of nurses, physicians, examinations, vaccinations, mobile teams, medical units, or other facilities required. Such calculations need explicit service standards, target coverage, morbidity and risk profiles, school-level enrollment and location, workforce productivity, current capacity, and geographic accessibility. Accordingly, any planning implications derived from the forecast should remain conditional: projected demographic growth may justify closer assessment of local capacity, while declining or geographically dispersed populations may still require evaluation against minimum access standards.

Several limitations remain. The study used official aggregated data and could not verify individual-level migration or cohort histories. Territorial labels were harmonized to the current 20-unit classification, while historical counts were used as reported in the official retrospective series; retrospective administrative revisions may therefore affect apparent transitions. The time-series comparisons were based on only 11 annual observations, the uncertainty analysis on five transition-year blocks, and the migration stress tests on 4 years of total net migration. The analysis did not include morbidity, service utilization, preventive-service coverage, workforce, infrastructure, costs, or school-level spatial distribution, and age-specific migration rates were unavailable. Future work should update the validation as new annual observations become available and link demographic projections to age-specific morbidity, service coverage and utilization, staffing norms, productivity, and travel-time measures before estimating resource requirements.

## 5 Conclusion

The age-shift cohort model had the lowest rolling-origin RMSE in 17 of 20 Kazakhstan territories over the 2021–2025 historical validation period and produced regionally heterogeneous projections of the population aged 6–17 years through 2030. Point estimates indicated the largest increases in Astana, Shymkent, Almaty city, and Mangystau Region and the largest decline in North Kazakhstan Region. After empirical transition-ratio uncertainty was considered, growth was supported in eight territories, decline in two, and direction remained uncertain in ten.

These forecasts quantify a demographic denominator rather than health-service need. They can serve as one input to regional planning when uncertainty is retained, but translating population projections into staffing requirements, service volumes, vaccination workload, or infrastructure needs requires additional information on morbidity, coverage, utilization, accessibility, productivity, service standards, and existing capacity.

## Data Availability

The raw data supporting the conclusions of this article will be made available by the authors, without undue reservation.
