# Supplementary material for: Forecasting Kazakhstan’s regional population aged 6–17 years to 2030: cohort validation and uncertainty analysis
Source: Front Public Health. 2026 Sep 7;14:1903413. doi: 10.3389/fpubh.2026.1903413 (PMC13590556; doi:10.3389/fpubh.2026.1903413)
Supplement: Supplementary file 1 [file Table_1.DOCX]

**Supplementary Materials**

**Supplementary statistical methods**

The focal age-shift cohort model, ARIMA, and ETS were evaluated on the same five expanding-window one-step targets for 2021–2025. At each rolling origin, cohort-transition coefficients were re-estimated from the five most recent annual transitions available strictly before the target year. The fixed 2024–2025 holdout was secondary; for the cohort model, coefficients were estimated from the five most recent transitions within the 2015–2023 training period and then held fixed across the two-step holdout projection. Model performance was summarized by mean absolute error (MAE), root mean square error (RMSE), mean absolute percentage error (MAPE), and mean error. The lowest regional RMSE defined the best-performing model within each validation design.

*MAE = (1/n) Σ |yᵢ − ŷᵢ|*

*RMSE = √[(1/n) Σ (yᵢ − ŷᵢ)²]*

*MAPE = (100/n) Σ |(yᵢ − ŷᵢ) / yᵢ|*

*Mean error = (1/n) Σ (yᵢ − ŷᵢ)*

Empirical transition-ratio uncertainty was quantified using 10,000 transition-year block-bootstrap simulations. Five transition years with origin years 2020–2024 were sampled with replacement as annual blocks; the same sampled blocks were applied to all coefficient cells within each simulation. The 10,000 repetitions stabilize empirical percentile estimates but do not increase the effective historical sample beyond the five observed annual transition blocks. The reported 2.5th and 97.5th percentiles are 95% empirical transition-ratio uncertainty limits, not comprehensive all-source prediction limits.

**Supplementary Table S1. Data inventory, derived analytical objects, and quality control**

| **Object** | **Period** | **Analytical grain** | **Variables / size** | **Role** |
| --- | --- | --- | --- | --- |
| Official population by single year of age | 2015–2025 | Year × territorial unit × sex × locality × age | Age 0–17; population count | Observed cohort cells and regional totals aged 6–17 |
| Official total regional population | 2021–2024 | Year × territorial unit | Total population | Consistency checks and school-age share for exploratory migration proxy |
| Official migration flows | 2021–2024 | Year × territorial unit | Arrivals, departures, net migration | Exploratory historical migration-effect stress tests |
| Observed regional population aged 6–17 | 2015–2025 | Year × 20 territorial units | Sum of single-year ages 6–17 | Forecast outcome and historical validation target |
| Cohort-transition ratios | Origin years 2020–2024 | Region × sex × locality × destination age | N(a+1,t+1)/N(a,t) | Median coefficients for focal 2026–2030 forecast |
| Rolling-origin predictions | Targets 2021–2025 | 20 units × 5 targets × 3 models | 300 predictions | Primary comparative validation |
| Fixed-holdout predictions | Targets 2024–2025 | 20 units × 2 targets × 3 models | 120 predictions | Secondary validation |
| Cohort forecasts | 2026–2030 | 20 units × 5 years | 100 region-year forecasts | Focal forecast |
| Block-bootstrap simulations | 2026–2030 | 10,000 simulations per region-year | 2.5th, 50th, 97.5th percentiles | Empirical transition-ratio uncertainty |

**Supplementary Table S1B. Quality-control results**

| **Check** | **Result** | **Status** |
| --- | --- | --- |
| Territorial units | 20 | PASS |
| Observed years | 2015-2025 | PASS |
| Age range | 0-17 | PASS |
| Conflicting duplicate keys | 0 | PASS |
| Identical duplicate rows removed | 0 | INFO |
| Observed national age 6-17 total in 2025 | 4491452 | PASS |
| Rolling-origin predictions | 300 | PASS |
| Fixed-holdout predictions | 120 | PASS |
| Cohort forecast region-years | 100 | PASS |
| Bootstrap simulations | 10000 | PASS |
| Missing core forecast values | 0 | PASS |
| Migration stress test | COMPLETED for 20 regions; exploratory only | PASS |

*All internal quality-control checks in the exported analytical package were PASS. The independent audit additionally reproduced all validation metrics and logical identities (28/28 checks).*

**Supplementary Table S2A. Region-specific rolling-origin accuracy for common one-step targets, 2021–2025**

| **Territorial unit** | **Model** | **n** | **MAE** | **RMSE** | **MAPE** | **Mean error** |
| --- | --- | --- | --- | --- | --- | --- |
| Abai Region | Cohort | 5 | 1,034.4 | 1,767.0 | 0.850% | −616.5 |
| Abai Region | ARIMA | 5 | 1,572.7 | 2,311.5 | 1.287% | −1,355.0 |
| Abai Region | ETS | 5 | 1,193.4 | 1,472.7 | 0.974% | +222.0 |
| Akmola Region | Cohort | 5 | 3,413.1 | 7,032.5 | 2.274% | +3,113.6 |
| Akmola Region | ARIMA | 5 | 4,631.5 | 6,734.3 | 3.076% | +2,735.3 |
| Akmola Region | ETS | 5 | 4,577.6 | 7,568.1 | 3.035% | +4,212.9 |
| Aktobe Region | Cohort | 5 | 1,431.5 | 2,851.2 | 0.727% | +1,218.1 |
| Aktobe Region | ARIMA | 5 | 4,017.9 | 4,700.7 | 1.990% | −1,008.0 |
| Aktobe Region | ETS | 5 | 6,388.1 | 7,992.8 | 3.218% | +4,368.5 |
| Almaty Region | Cohort | 5 | 4,148.2 | 6,977.3 | 1.215% | +3,330.7 |
| Almaty Region | ARIMA | 5 | 5,727.9 | 7,731.6 | 1.636% | +822.1 |
| Almaty Region | ETS | 5 | 10,711.2 | 14,765.7 | 3.109% | +8,404.9 |
| Almaty city | Cohort | 5 | 11,211.5 | 19,188.6 | 2.883% | +6,194.8 |
| Almaty city | ARIMA | 5 | 14,178.4 | 19,858.1 | 3.656% | +4,497.5 |
| Almaty city | ETS | 5 | 19,661.2 | 29,722.9 | 5.084% | +19,661.2 |
| Astana | Cohort | 5 | 4,199.1 | 4,650.0 | 1.531% | +1,332.9 |
| Astana | ARIMA | 5 | 5,723.7 | 6,549.4 | 2.128% | +4,238.6 |
| Astana | ETS | 5 | 12,014.7 | 14,715.7 | 4.549% | +12,014.7 |
| Atyrau Region | Cohort | 5 | 626.3 | 847.9 | 0.374% | +19.2 |
| Atyrau Region | ARIMA | 5 | 1,643.6 | 1,863.8 | 0.964% | −1,018.8 |
| Atyrau Region | ETS | 5 | 3,260.0 | 4,270.8 | 1.965% | +889.5 |
| East Kazakhstan Region | Cohort | 5 | 515.2 | 888.6 | 0.430% | +515.2 |
| East Kazakhstan Region | ARIMA | 5 | 1,137.4 | 1,315.3 | 0.931% | −669.2 |
| East Kazakhstan Region | ETS | 5 | 1,854.0 | 2,421.6 | 1.524% | −255.9 |
| Karaganda Region | Cohort | 5 | 595.1 | 711.1 | 0.287% | +401.1 |
| Karaganda Region | ARIMA | 5 | 1,513.5 | 1,940.8 | 0.718% | −1,268.5 |
| Karaganda Region | ETS | 5 | 2,989.8 | 3,566.6 | 1.426% | −912.4 |
| Kostanay Region | Cohort | 5 | 498.3 | 791.0 | 0.355% | +204.4 |
| Kostanay Region | ARIMA | 5 | 911.8 | 1,048.6 | 0.647% | −462.8 |
| Kostanay Region | ETS | 5 | 1,436.7 | 1,737.0 | 1.021% | +66.3 |
| Kyzylorda Region | Cohort | 5 | 688.8 | 748.5 | 0.330% | −251.6 |
| Kyzylorda Region | ARIMA | 5 | 1,885.8 | 2,237.9 | 0.893% | −1,552.2 |
| Kyzylorda Region | ETS | 5 | 3,068.2 | 3,394.3 | 1.465% | −584.3 |
| Mangystau Region | Cohort | 5 | 1,538.2 | 2,681.7 | 0.821% | +1,122.5 |
| Mangystau Region | ARIMA | 5 | 3,245.9 | 3,651.4 | 1.700% | −358.7 |
| Mangystau Region | ETS | 5 | 6,201.5 | 7,967.4 | 3.273% | +4,727.1 |
| North Kazakhstan Region | Cohort | 5 | 784.4 | 1,369.4 | 0.884% | −543.1 |
| North Kazakhstan Region | ARIMA | 5 | 1,085.2 | 1,699.4 | 1.224% | −911.2 |
| North Kazakhstan Region | ETS | 5 | 874.9 | 1,298.0 | 0.987% | −701.0 |
| Pavlodar Region | Cohort | 5 | 692.3 | 1,153.2 | 0.512% | +564.1 |
| Pavlodar Region | ARIMA | 5 | 1,553.5 | 1,740.5 | 1.126% | −851.5 |
| Pavlodar Region | ETS | 5 | 2,674.9 | 3,175.5 | 1.945% | +180.5 |
| Shymkent | Cohort | 5 | 3,807.3 | 5,538.3 | 1.363% | +848.6 |
| Shymkent | ARIMA | 5 | 5,314.1 | 6,589.0 | 1.882% | +11.4 |
| Shymkent | ETS | 5 | 9,514.1 | 12,547.1 | 3.407% | +5,610.3 |
| Turkistan Region | Cohort | 5 | 3,610.3 | 4,691.6 | 0.615% | +2,485.5 |
| Turkistan Region | ARIMA | 5 | 6,627.6 | 7,116.6 | 1.109% | −3,267.8 |
| Turkistan Region | ETS | 5 | 10,362.6 | 12,327.5 | 1.745% | −799.8 |
| Ulytau Region | Cohort | 5 | 331.2 | 568.5 | 0.707% | −255.3 |
| Ulytau Region | ARIMA | 5 | 444.1 | 657.1 | 0.943% | −281.8 |
| Ulytau Region | ETS | 5 | 741.6 | 815.0 | 1.548% | +360.4 |
| West Kazakhstan Region | Cohort | 5 | 665.8 | 1,088.0 | 0.501% | +282.3 |
| West Kazakhstan Region | ARIMA | 5 | 1,374.6 | 1,488.3 | 1.014% | −407.5 |
| West Kazakhstan Region | ETS | 5 | 2,306.2 | 3,078.6 | 1.722% | +1,288.6 |
| Zhambyl Region | Cohort | 5 | 2,510.4 | 3,867.4 | 0.843% | +1,802.8 |
| Zhambyl Region | ARIMA | 5 | 4,633.7 | 5,382.4 | 1.528% | −2,386.8 |
| Zhambyl Region | ETS | 5 | 6,699.6 | 8,996.4 | 2.219% | −691.0 |
| Zhetisu Region | Cohort | 5 | 1,544.6 | 2,513.1 | 0.995% | +1,309.8 |
| Zhetisu Region | ARIMA | 5 | 2,395.2 | 2,749.6 | 1.521% | −567.8 |
| Zhetisu Region | ETS | 5 | 3,684.6 | 4,679.9 | 2.344% | +1,117.5 |

Note: Five common one-step targets were evaluated for each territorial unit and model. At each origin, cohort-transition coefficients were re-estimated from the five most recent annual transitions available strictly before the target year.

**Supplementary Table S2B. Region-specific fixed-holdout accuracy, 2024–2025**

| **Territorial unit** | **Model** | **n** | **MAE** | **RMSE** | **MAPE** | **Mean error** |
| --- | --- | --- | --- | --- | --- | --- |
| Abai Region | Cohort | 2 | 156.0 | 187.2 | 0.126% | −103.4 |
| Abai Region | ARIMA | 2 | 1,693.5 | 1,934.2 | 1.365% | −1,693.5 |
| Abai Region | ETS | 2 | 981.2 | 982.1 | 0.791% | +981.2 |
| Akmola Region | Cohort | 2 | 281.7 | 333.1 | 0.181% | +281.7 |
| Akmola Region | ARIMA | 2 | 3,627.5 | 3,875.6 | 2.329% | −3,627.5 |
| Akmola Region | ETS | 2 | 2,396.7 | 2,481.6 | 1.540% | +2,396.7 |
| Aktobe Region | Cohort | 2 | 463.7 | 467.2 | 0.218% | −463.7 |
| Aktobe Region | ARIMA | 2 | 3,886.5 | 4,092.6 | 1.820% | −3,886.5 |
| Aktobe Region | ETS | 2 | 3,853.0 | 4,057.3 | 1.804% | −3,853.0 |
| Almaty Region | Cohort | 2 | 1,294.8 | 1,700.9 | 0.346% | +1,294.8 |
| Almaty Region | ARIMA | 2 | 7,364.6 | 7,798.9 | 1.983% | −7,364.6 |
| Almaty Region | ETS | 2 | 2,531.9 | 3,166.7 | 0.678% | −1,902.0 |
| Almaty city | Cohort | 2 | 4,139.3 | 4,523.4 | 0.974% | −4,139.3 |
| Almaty city | ARIMA | 2 | 8,789.7 | 9,421.0 | 2.071% | −8,789.7 |
| Almaty city | ETS | 2 | 24,415.6 | 25,594.3 | 5.761% | +24,415.6 |
| Astana | Cohort | 2 | 7,557.8 | 8,908.4 | 2.467% | +7,557.8 |
| Astana | ARIMA | 2 | 7,629.1 | 9,047.0 | 2.489% | +7,629.1 |
| Astana | ETS | 2 | 8,934.9 | 10,053.5 | 2.932% | +8,934.9 |
| Atyrau Region | Cohort | 2 | 775.8 | 968.4 | 0.440% | −775.8 |
| Atyrau Region | ARIMA | 2 | 3,472.0 | 3,853.6 | 1.975% | −3,472.0 |
| Atyrau Region | ETS | 2 | 3,271.0 | 3,701.9 | 1.860% | −3,271.0 |
| East Kazakhstan Region | Cohort | 2 | 114.7 | 133.9 | 0.093% | +114.7 |
| East Kazakhstan Region | ARIMA | 2 | 2,629.3 | 2,847.7 | 2.131% | −2,629.3 |
| East Kazakhstan Region | ETS | 2 | 2,806.1 | 3,011.3 | 2.275% | −2,806.1 |
| Karaganda Region | Cohort | 2 | 191.6 | 227.3 | 0.090% | −191.6 |
| Karaganda Region | ARIMA | 2 | 4,529.3 | 4,829.3 | 2.137% | −4,529.3 |
| Karaganda Region | ETS | 2 | 5,184.3 | 5,470.6 | 2.446% | −5,184.3 |
| Kostanay Region | Cohort | 2 | 428.7 | 481.9 | 0.303% | −428.7 |
| Kostanay Region | ARIMA | 2 | 1,777.2 | 1,993.8 | 1.256% | −1,777.2 |
| Kostanay Region | ETS | 2 | 1,851.9 | 2,066.1 | 1.309% | −1,851.9 |
| Kyzylorda Region | Cohort | 2 | 941.1 | 1,114.7 | 0.437% | −941.1 |
| Kyzylorda Region | ARIMA | 2 | 4,339.7 | 4,828.3 | 2.014% | −4,339.7 |
| Kyzylorda Region | ETS | 2 | 5,553.6 | 5,971.9 | 2.579% | −5,553.6 |
| Mangystau Region | Cohort | 2 | 773.7 | 840.7 | 0.372% | −773.7 |
| Mangystau Region | ARIMA | 2 | 2,392.0 | 2,631.6 | 1.149% | −2,392.0 |
| Mangystau Region | ETS | 2 | 2,449.9 | 2,692.2 | 1.177% | −2,449.9 |
| North Kazakhstan Region | Cohort | 2 | 172.5 | 189.3 | 0.195% | −77.9 |
| North Kazakhstan Region | ARIMA | 2 | 522.0 | 656.4 | 0.592% | −522.0 |
| North Kazakhstan Region | ETS | 2 | 522.0 | 656.4 | 0.592% | −522.0 |
| Pavlodar Region | Cohort | 2 | 158.4 | 216.6 | 0.113% | −147.7 |
| Pavlodar Region | ARIMA | 2 | 3,188.9 | 3,487.3 | 2.286% | −3,188.9 |
| Pavlodar Region | ETS | 2 | 3,493.1 | 3,784.5 | 2.504% | −3,493.1 |
| Shymkent | Cohort | 2 | 3,312.7 | 3,424.5 | 1.108% | −3,312.7 |
| Shymkent | ARIMA | 2 | 6,662.2 | 7,039.0 | 2.225% | −6,662.2 |
| Shymkent | ETS | 2 | 5,476.1 | 6,002.2 | 1.826% | −5,476.1 |
| Turkistan Region | Cohort | 2 | 1,932.0 | 2,391.3 | 0.317% | −1,932.0 |
| Turkistan Region | ARIMA | 2 | 12,635.5 | 13,778.0 | 2.076% | −12,635.5 |
| Turkistan Region | ETS | 2 | 12,817.1 | 13,965.6 | 2.106% | −12,817.1 |
| Ulytau Region | Cohort | 2 | 132.3 | 167.7 | 0.269% | −132.3 |
| Ulytau Region | ARIMA | 2 | 241.6 | 316.1 | 0.492% | −241.6 |
| Ulytau Region | ETS | 2 | 1,216.1 | 1,248.4 | 2.486% | +1,216.1 |
| West Kazakhstan Region | Cohort | 2 | 445.9 | 487.1 | 0.314% | −445.9 |
| West Kazakhstan Region | ARIMA | 2 | 1,644.8 | 1,759.2 | 1.161% | −1,644.8 |
| West Kazakhstan Region | ETS | 2 | 1,759.1 | 1,879.6 | 1.241% | −1,759.1 |
| Zhambyl Region | Cohort | 2 | 1,151.2 | 1,369.1 | 0.375% | −1,151.2 |
| Zhambyl Region | ARIMA | 2 | 10,647.8 | 11,505.2 | 3.473% | −10,647.8 |
| Zhambyl Region | ETS | 2 | 10,396.4 | 11,265.8 | 3.391% | −10,396.4 |
| Zhetisu Region | Cohort | 2 | 249.3 | 308.0 | 0.155% | −180.8 |
| Zhetisu Region | ARIMA | 2 | 4,395.4 | 4,815.8 | 2.734% | −4,395.4 |
| Zhetisu Region | ETS | 2 | 3,473.7 | 3,970.9 | 2.160% | −3,473.7 |

Note: The training period was 2015–2023 and the test period was 2024–2025. For the cohort model, coefficients were estimated within the training period and held fixed across the two-step holdout projection. The fixed holdout is secondary because it contains fewer forecast origins.

**Supplementary Table S2C. Model selection under the two validation designs**

| **Territorial unit** | **Best model: rolling-origin** | **Best model: fixed holdout** | **Agreement** |
| --- | --- | --- | --- |
| Abai Region | ETS | Cohort | No |
| Akmola Region | ARIMA | Cohort | No |
| Aktobe Region | Cohort | Cohort | Yes |
| Almaty Region | Cohort | Cohort | Yes |
| Almaty city | Cohort | Cohort | Yes |
| Astana | Cohort | Cohort | Yes |
| Atyrau Region | Cohort | Cohort | Yes |
| East Kazakhstan Region | Cohort | Cohort | Yes |
| Karaganda Region | Cohort | Cohort | Yes |
| Kostanay Region | Cohort | Cohort | Yes |
| Kyzylorda Region | Cohort | Cohort | Yes |
| Mangystau Region | Cohort | Cohort | Yes |
| North Kazakhstan Region | ETS | Cohort | No |
| Pavlodar Region | Cohort | Cohort | Yes |
| Shymkent | Cohort | Cohort | Yes |
| Turkistan Region | Cohort | Cohort | Yes |
| Ulytau Region | Cohort | Cohort | Yes |
| West Kazakhstan Region | Cohort | Cohort | Yes |
| Zhambyl Region | Cohort | Cohort | Yes |
| Zhetisu Region | Cohort | Cohort | Yes |

*Rolling-origin best model: cohort 17, ETS 2, ARIMA 1. Fixed holdout best model: cohort 20, ETS 0, ARIMA 0. Selection agreed in 17 of 20 territorial units.*

**Supplementary Table S3. Sensitivity of the 2030 cohort forecast to coefficient specification**

| **Territorial unit** | **Focal median 2020–2024** | **Minimum across specifications** | **Maximum across specifications** | **Range** |
| --- | --- | --- | --- | --- |
| Abai Region | 120,364 | 117,475 | 120,527 | 3,052 |
| Akmola Region | 145,059 | 144,052 | 164,460 | 20,408 |
| Aktobe Region | 233,445 | 232,035 | 242,429 | 10,395 |
| Almaty Region | 383,308 | 383,308 | 408,425 | 25,117 |
| Almaty city | 519,727 | 509,656 | 573,327 | 63,671 |
| Astana | 412,128 | 412,128 | 428,869 | 16,740 |
| Atyrau Region | 190,441 | 189,788 | 192,014 | 2,226 |
| East Kazakhstan Region | 118,879 | 117,896 | 121,251 | 3,355 |
| Karaganda Region | 208,429 | 206,956 | 209,150 | 2,195 |
| Kostanay Region | 132,892 | 132,221 | 134,967 | 2,746 |
| Kyzylorda Region | 224,399 | 223,816 | 224,399 | 583 |
| Mangystau Region | 242,669 | 239,503 | 250,208 | 10,705 |
| North Kazakhstan Region | 78,254 | 76,726 | 78,391 | 1,666 |
| Pavlodar Region | 133,422 | 132,768 | 134,815 | 2,047 |
| Shymkent | 372,632 | 365,188 | 385,310 | 20,123 |
| Turkistan Region | 604,463 | 604,463 | 616,176 | 11,712 |
| Ulytau Region | 50,043 | 49,397 | 50,363 | 966 |
| West Kazakhstan Region | 150,421 | 150,059 | 153,449 | 3,390 |
| Zhambyl Region | 300,197 | 298,654 | 308,037 | 9,383 |
| Zhetisu Region | 156,665 | 155,663 | 163,120 | 7,457 |

*Specifications: median 2020–2024 (focal), mean 2020–2024, median 2022–2024, and median 2018–2024. Range = maximum minus minimum forecast across the four specifications.*

**Supplementary Table S4. Exploratory historical migration-effect stress tests for 2030**

| **Territorial unit** | **Annual proxy** | **Attenuated 0×** | **Baseline 1×** | **Amplified 2×** | **Scenario range** |
| --- | --- | --- | --- | --- | --- |
| Abai Region | −1,513.4 | 127,931 | 120,364 | 112,797 | 15,134 |
| Akmola Region | −711.0 | 148,614 | 145,059 | 141,503 | 7,110 |
| Aktobe Region | −481.1 | 235,850 | 233,445 | 231,039 | 4,811 |
| Almaty Region | +553.7 | 380,540 | 383,308 | 386,077 | 5,537 |
| Almaty city | +6,660.6 | 486,424 | 519,727 | 553,030 | 66,606 |
| Astana | +9,287.0 | 365,694 | 412,128 | 458,563 | 92,870 |
| Atyrau Region | −655.8 | 193,720 | 190,441 | 187,162 | 6,558 |
| East Kazakhstan Region | −628.6 | 122,022 | 118,879 | 115,736 | 6,286 |
| Karaganda Region | −1,099.8 | 213,928 | 208,429 | 202,930 | 10,998 |
| Kostanay Region | −803.5 | 136,909 | 132,892 | 128,874 | 8,035 |
| Kyzylorda Region | −1,716.6 | 232,982 | 224,399 | 215,816 | 17,166 |
| Mangystau Region | +616.2 | 239,588 | 242,669 | 245,750 | 6,162 |
| North Kazakhstan Region | −832.9 | 82,419 | 78,254 | 74,089 | 8,329 |
| Pavlodar Region | −841.5 | 137,629 | 133,422 | 129,214 | 8,415 |
| Shymkent | +1,592.6 | 364,669 | 372,632 | 380,595 | 15,926 |
| Turkistan Region | −6,500.9 | 636,968 | 604,463 | 571,959 | 65,009 |
| Ulytau Region | −532.6 | 52,706 | 50,043 | 47,380 | 5,326 |
| West Kazakhstan Region | −418.1 | 152,512 | 150,421 | 148,330 | 4,181 |
| Zhambyl Region | −3,183.0 | 316,112 | 300,197 | 284,282 | 31,830 |
| Zhetisu Region | −2,036.2 | 166,846 | 156,665 | 146,484 | 20,362 |

*The annual proxy equals mean total net migration in 2021–2024 multiplied by the mean population share aged 6–17 years. The 0× and 2× alternatives are stylized stress tests around the historical effect already embedded in cohort-transition ratios; they are not probabilistic forecasts of child migration.*
